# 753. Impact of COVID-19 on Healthcare Facility-Onset *Clostridiodes difficile* Infection

**DOI:** 10.1093/ofid/ofab466.950

**Published:** 2021-12-04

**Authors:** Geehan Suleyman, Rafa Khansa, Ramesh Mayur, Indira Brar, Rachel Kenney

**Affiliations:** Henry Ford Hospital, Detroit, Michigan

## Abstract

**Background:**

It is estimated that the majority of hospitalized COVID-19 patients around the world received antibiotics despite the fact that bacterial co-infections are rare. This can lead to increased antimicrobial resistance and *Clostridioides difficile* infections (CDI). Gastrointestinal symptoms of COVID-19 may also contribute to increased testing. The objective of this study was to assess the impact of the COVID-19 pandemic on our healthcare facility-onset (HO) CDI rates.

**Methods:**

This was a retrospective cross-sectional study comparing CDI rate per 1,000 patient days, *C. diff* order rate per 1,000 patient days, Standardized Antimicrobial Administration Ratio (SAAR), and Standardized Infection Ratio (SIR) in the pre-COVID-19 period from January 1, 2019 to December 31, 2019 to the COVID-19 period from April 1, 2020 to March 31, 2021 at a 877-bed tertiary care hospital in Detroit, Michigan. CDI and order rates were extracted from the electronic medical record (Epic™ Bugsy). SAAR and SIR data were extracted from National Healthcare Safety Network (NHSN).

**Results:**

The average CDI rate per 1,000 patient days was 4.29 pre-COVID-19 compared to 1.98 during COVID-19 with a 54% reduction, and the *C. diff* order rate per 1,000 patient days also decreased from 130.89 to 93.03, resulting in a 29% reduction (Figure 1). The SIR was 0.383 compared to 0.308 during COVID-19 (P-value 0.404). SAAR decreased from 1.095 to 0.945 (P-value < 0.001). However, our institution experienced three COVID-19 waves, with peaks in April 2020, November 2020 and March 2021, that correlated with high risk CDI antibiotic utilization in intensive care unit (ICU) (Figure 2). The average hand hygiene rate increased from 82% to 92%.

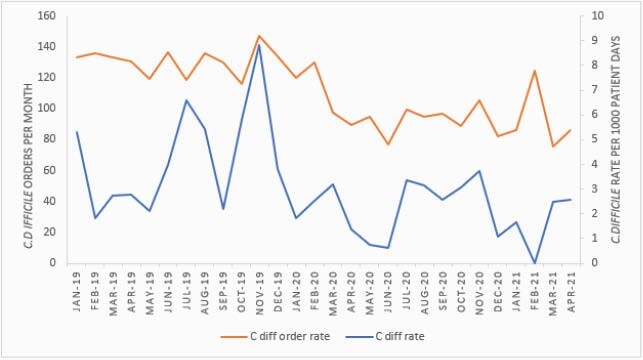

Figure 1. Clostridioides difficile order and infection rates pre-and during COVID-19 pandemic.

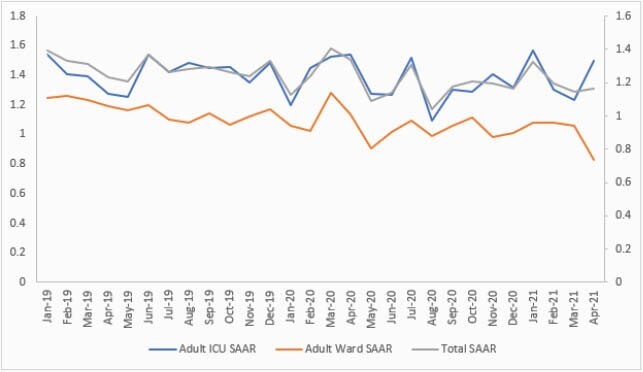

Figure 2. Standardized Antimicrobial Administration Ratio (SAAR) pre-and during COVID-19 pandemic.

**Conclusion:**

Despite the COVID-19 pandemic, the HO-CDI and *C. diff* order rates and overall SAAR decreased; however, antibiotic utilization increased in the ICU during the COVID-19 waves. The overall decrease may be multifactorial and related to increased hand hygiene compliance, isolation and personal protective equipment use and overall decreased antibiotic use and *C. diff* orders.

**Disclosures:**

**Rachel Kenney, PharmD**, **Medtronic, Inc.** (Other Financial or Material Support, spouse is an employee and shareholder)

